# Intrapopulation Variability Shaping Isotope Discrimination and Turnover: Experimental Evidence in Arctic Foxes

**DOI:** 10.1371/journal.pone.0021357

**Published:** 2011-06-23

**Authors:** Nicolas Lecomte, Øystein Ahlstrøm, Dorothée Ehrich, Eva Fuglei, Rolf A. Ims, Nigel G. Yoccoz

**Affiliations:** 1 Department of Arctic and Marine Biology, University of Tromsø, Tromsø, Norway; 2 Department of Animal and Aquacultural Sciences, Norwegian University of Life Science, Ås, Norway; 3 Norwegian Polar Institute, Fram Center, Tromsø, Norway; Institut Pluridisciplinaire Hubert Curien, France

## Abstract

**Background:**

Tissue-specific stable isotope signatures can provide insights into the trophic ecology of consumers and their roles in food webs. Two parameters are central for making valid inferences based on stable isotopes, isotopic discrimination (difference in isotopic ratio between consumer and its diet) and turnover time (renewal process of molecules in a given tissue usually measured when half of the tissue composition has changed). We investigated simultaneously the effects of age, sex, and diet types on the variation of discrimination and half-life in nitrogen and carbon stable isotopes (δ^15^N and δ^13^C, respectively) in five tissues (blood cells, plasma, muscle, liver, nail, and hair) of a top predator, the arctic fox *Vulpes lagopus*.

**Methodology/Principal Findings:**

We fed 40 farmed foxes (equal numbers of adults and yearlings of both sexes) with diet capturing the range of resources used by their wild counterparts. We found that, for a single species, six tissues, and three diet types, the range of discrimination values can be almost as large as what is known at the scale of the whole mammalian or avian class. Discrimination varied depending on sex, age, tissue, and diet types, ranging from 0.3‰ to 5.3‰ (mean  = 2.6‰) for δ^15^N and from 0.2‰ to 2.9‰ (mean  = 0.9‰) for δ^13^C. We also found an impact of population structure on δ^15^N half-life in blood cells. Varying across individuals, δ^15^N half-life in plasma (6 to 10 days) was also shorter than for δ^13^C (14 to 22 days), though δ^15^N and δ^13^C half-lives are usually considered as equal.

**Conclusion/Significance:**

Overall, our multi-factorial experiment revealed that at least six levels of isotopic variations could co-occur in the same population. Our experimental analysis provides a framework for quantifying multiple sources of variation in isotopic discrimination and half-life that needs to be taken into account when designing and analysing ecological field studies.

## Introduction

Measuring nutritional flows between consumers and their resources plays a central role in the understanding of trophic interactions and energy pathways. One way to conduct such measurement relies on the use of naturally occurring, stable isotopes. The rapid development in stable isotopes studies has fuelled our perspectives in functional ecology, allowing, in particular, the reconstruction of diet from different food types, assimilated over various windows of time (e.g. [1]). Despite the wide application of this tool in ecological field studies, more validation with experimental studies under controlled conditions is needed [2], [3]. Alternatively, researchers are bound to rely on meta-analyses with their inherent limitations [4]. Thus, the sources of variation in isotopic composition among consumers are still imperfectly understood, though they have raised fundamental and applied perspective for monitoring nutritional flows.

Two parameters are central to the accurate use of stable isotopes in ecology [3], [4], [5], [6], isotopic discrimination (difference in isotopes ratios between a consumer and its diet) and turnover time (renewal process of stable isotope in a given tissue, which is usually represented by the half-life, the time required for a 50% turnover (following the original definition of turnover; [7], [8])). Correct estimates of discrimination are a prerequisite to describe trophic interactions in a robust manner and measuring turnover allows identifying the time window for which change in diet can be tracked. In diet reconstruction approaches, isotopic mixing models are sensitive to the uncertainty and bias in discrimination estimates [9]. Despite the possibility to incorporate uncertainty of trophic discrimination factors in the new, Bayesian mixing models (e.g. [10]), this uncertainty can be large [11], especially when using values from meta-analyses or experiments with low sample size (e.g. [4]).

So far, the experimental studies of discrimination have shown evidence for variations attributable to age, dietary ontogeny, nutritional state, body size, or diet composition [1], [3], [9], [12], [13]. For the stable isotope ratios of carbon (noted δ^13^C) and nitrogen (δ^15^N), the two most commonly used in trophic ecology, five experimental studies also found that variance was due to the isotopic value of the diet eaten [2], [12], [14], [15], [16], though difference in nutritional quality between diet were not accounted for [4], [17]. Considering whole body, there is usually only a small enrichment in δ^13^C for each trophic level (0.5‰ –1‰) though large variation exists among tissues (e.g. [1]). For δ^15^N, a mean discrimination factor of 3.5‰ is the rule of thumb used for diet analyses [18]. It is worth mentioning that several studies have developed strategies for measuring discrimination on free-ranging animals (e.g. [19]), though there is still a debate as to how much uncontrolled conditions and variability in natural diet affect the measurements of discrimination [3], [4], [20].

Isotopic turnover can vary from a few days up to several months for different tissues within a single species [1], [3], [5]. The variance of turnover across tissues can be used to monitor diet at different temporal windows, but also across spatial scales when the target species change its location during the course of its life cycle. The choice of tissue to analyse should then be directed by the ecological question as well as the feasibility of sampling (e.g. [1], [21]). Possible underlying factors influencing isotopic turnover are body size, growth, temperature, and protein turnover (review in [3], [22]). While it can be assumed that δ^13^C and δ^15^N have similar turnover rates [3], [23], two experimental studies have clearly demonstrated that it can differ by two orders of magnitude [2], [24]. It confirms that such variability can indeed be tissue-specific [25].

Despite the numerous efforts to identify sources of variation in discrimination and tissue turnover, several variables have not yet been investigated in detail, as for instance the effect of sex. In addition, individual heterogeneity has not previously been accounted for in the statistical modelling, despite being an important issue in ecophysiological research. Though the number of experimental studies to understand stable isotope ecology is increasing [3], the frame of experimental designs is often restricted to only one sex or one age class used to draw population or species level inference. In addition, many studies used small sample sizes, (median = 5; min  = 3; max  = 75; n = 65 published papers; See Supporting Information from Caut et al. [26]), with, for instance, only one individual for one sex only (Lecomte Nicolas et al., unpublished data). This is likely due to several, non exclusive reasons, for instance to past logistical and financial limitations for batch processing in the laboratory, to restriction linked to destructive sampling as well as the ability to maintain stocks of large animals in captivity. Such restricted sampling frame may bias analyses of free-ranging animals since group proportions are dependent on population structure and the method of sampling tissues. Such small sample sizes may also have hampered the ability to test for multiple effects (e.g. sex, age and diet) within the same study. Though these effects are already described for several species, we do not know whether they can occur simultaneously and act perhaps synergistically (i.e. interact) in the very same population and how to rank them in terms of impact on discrimination factors.

**Figure 1:**
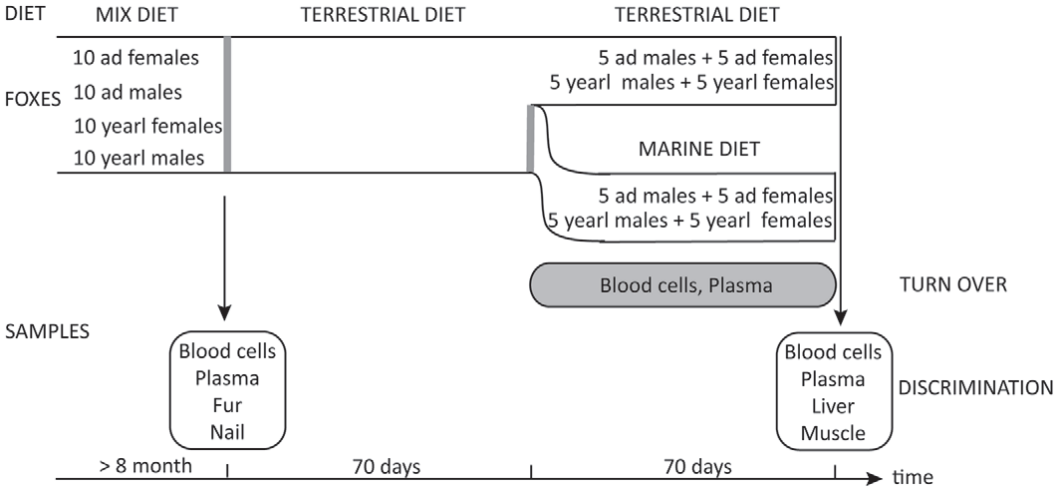
Experimental scheme for analysing diet (mix, terrestrial, and marine) shifts and the stable isotopes of various tissues samples in arctic foxes. Ad and Yearl correspond to adults and yearlings, respectively.

Our purpose here was to quantify the importance of intra-population variability, i.e. age, sex and individual component, for diet-tissue discrimination and turnover and how it can interact with tissues and diet types with varying isotopic composition. Our relatively large sample size (40 individuals) provides the opportunity to assess simultaneously the effects of age, diet, sex, and individual variability. We used farmed arctic foxes (*Vulpes lagopus*), a top predator species, which can in the wild switch between diet types covering a large range of isotopic values, such as marine and terrestrial resources (e.g. [27]). We targeted tissues known in the literature for their contrasting discrimination and turnover values (Figure 1). We aimed to identify which tissues have low variability in discrimination and turnover values, while accounting for the relative impact of population structure and diet characteristic. As such, these tissues could thus be recommended as providing the most precise diet estimates in future field studies. We also carried out simulations to assess the reliability of turnover measurement, with one and two compartment models (Cerling et al. 2007; Carleton et al. 2008; Martinez del Rio & Anderson-Sprecher 2008). Our study ought to provide insights for both further experimental studies as well as field investigations based on stable isotopes.

## Results

### Isotopic discrimination

**Figure 2:**
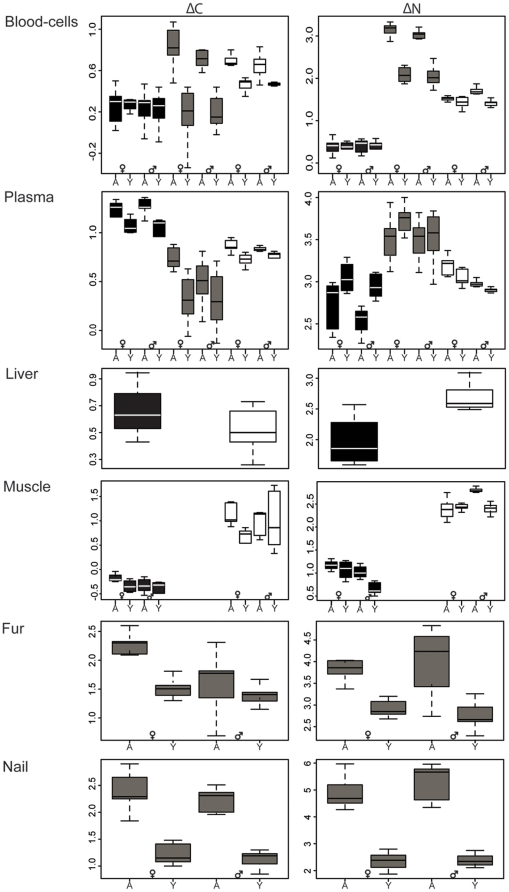
Discrimination (Δ) for both ^13^C and ^15^N isotopes for various tissues, diet, and population components in arctic foxes. The marine, mix and terrestrial diet are represented in black, grey, and white boxplots, respectively. A and Y correspond to adults and yearlings, respectively with a total sample size of 40. See Material and Methods for details.

Isotopic discrimination showed great variability among the experimental groups based on diet types, tissues, sex, and age, ranging from 0.3‰ to 5.3‰ (mean ± SD  = 2.6±0.4) for δ^15^N and from 0.2‰ to 2.9‰ for δ^13^C (mean ± SD  = 0.9±0.2) (Table 1; Figure 2). For blood cells and plasma, the selected mixed model included all fixed effects (diet, tissue, age, sex) and all two-way interactions. Confidence intervals of the parameter estimates showed that there was a significant effect of diet on Δ^15^N only, but that there was a significant interaction of diet with age for both isotopes (Table S1). For the mix diet, discrimination in yearlings was higher than in adults. Compared to plasma, Δ^13^C and Δ^15^N in blood cells were on average 0.3‰ and 1.5‰ lower, respectively.

**Table 1:** Mean discrimination factors ± SD (‰) between experimental diet and tissues of arctic foxes.

			Tissues					
			Active				Inactive	
	Sex	Age	Blood cells	Plasma	Liver	Muscle	Fur	Nail
ΔC	F	Y	0.28±0.16	0.59±0.25	-	0.23±0.60	1.98±0.16	1.72±0.32
		A	0.67±0.19	0.88±0.16	-	0.64±0.79	2.65±0.22	2.94±0.32
	M	Y	0.27±0.13	0.61±0.25	-	0.32±0.85	1.89±0.13	1.58±0.41
		A	0.57±0.19	0.80±0.28	-	0.31±0.70	2.16±0.32	2.67±0.58
	Overall		0.45±0.12 (17)	0.72±0.18 (21)	0.57±0.19 (10)	0.37±0.76 (40)	2.18±0.44 (39)	2.19±0.64 (34)
	Overall Mix		0.49±0.25 (17)	0.47±0.16 (17)	-	-	2.18±0.44 (39)	2.19±0.64 (34)
	Overall Mar		0.24±0.16 (17)	1.15±0.12(17)	0.66±0.22 (4)*	-0.32±0.13 (20)*	-	-
	Overall Terr		0.56±0.12 (17)	0.79±0.08 (17)	0.51±0.22(10)*	0.95±0.36(20)*	-	-
ΔN	F	Y	1.55±0.82	3.41±0.28	-	1.83±0.76	2.85±0.25	2.15±0.32
		A	2.09±0.54	3.24±0.28	-	1.94±0.73	3.79±0.38	4.70±0.85
	M	Y	1.46±0.85	3.24±0.28	-	1.59±0.85	2.77±0.32	2.47±0.38
		A	1.93±0.85	3.08±0.35	-	1.92±0.95	4.03±0.54	5.29±0.82
	Overall		1.76±0.45 (17)	3.25±0.23 (21)	2.40±0.47 (10)	1.79±0.41 (40)	3.34±0.69 (39)	3.60±0.73 (34)
	Overall Mix		2.56±0.37 (17)	3.57±0.16 (17)	-	-	3.34±0.69 (39)	3.60±0.73 (34)
	Overall Mar		0.38±0.16 (17)	2.80±0.29 (17)	1.97±0.44 (4)*	0.95±0.19 (20)*	-	-
	Overall Terr		1.56±0.12 (17)	3.03±0.12 (17)	2.68±0.28 (10)*	2.51±0.16 (20)*	-	-

Subscripts: Sample sizes are within parentheses. ΔC are obtained after lipid correction (see details in methods). A: adult; F: female; M: male; Y: yearling;

*assuming that the delay since diet shift was long enough to exceed the turnover of both liver and muscle, following other estimates available from other mammals [5]. There was missing information for some individuals, which resulted in varying sample sizes in the different analyses or summary estimates.

For the liver samples, Δ^15^N was 0.7‰ lower in the marine diet compared to the terrestrial one (Tables S1 & S2). For muscle samples, Δ^13^C was higher in adults compared to yearlings, though no such effect was found for Δ^15^N. However, for both isotopes, discrimination varied differently between adults and yearlings within each sex. Δ^13^C was indeed higher for female yearlings though in Δ^15^N, this was only true for male yearlings (Tables S1 & S2).

For fur and nail, discrimination for both isotopes differed between ages (Table S1), being ca 1.3‰ higher for adults on average (Table 1). Age effect also differed according to sex for Δ^13^C and tissue in both isotopes (Table S1 & Figure 2). In addition, we also detected a Δ^13^C ca 0.7‰ higher in fur compared to nails.

Overall, sex and age effects appeared tissue-, diet-, and isotope- specific. These factors had the most important impact in fur and nail for both Δ^13^C and Δ^15^N. In blood and muscle, we found a somewhat complex impact of sex, age, tissue, and their interactions, which were isotope dependent.

### Isotopic turnover

For both plasma and blood cells, two-compartment models failed to converge using both simple non-linear least squares and non-linear mixed models. This failure to do so is often an indication of overparametrization (see the method section in [28]). The same problems were encountered using Bayesian approaches and other parameterizations (see methods for details), further supporting the one-compartment model for our data.

**Figure 3:**
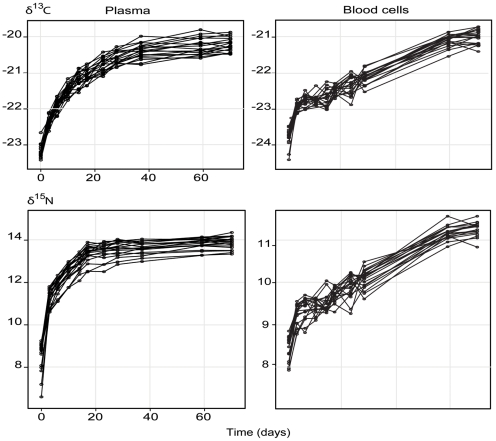
Stable-carbon/nitrogen isotopes' turnover for plasma and blood cells of arctic foxes. Turnover are illustrated with one line trajectories per individual (n = 20 corresponding to animals shifted from a terrestrial diet to a marine one). Model estimates of isotopic half-lives are provided in Table S4.

Using the one-compartment model, the isotopic values of the group remaining on the terrestrial diet (Figure 1) did not change between day 0 up until the end of the experiment (Figure S1), supporting their status as control group (equal initial and asymptotic values). This also supported that isotopic equilibrium was reached for plasma and blood cells before the shift to marine diet. Further evidence of equilibrium was obtained for plasma in the marine group after 70 days with a clear plateau (Figure 3). The plateau was not obvious for blood cells as individual variation was larger than in plasma. However, if we model the turnover of blood cells without accounting for individual identity (i.e. in the way usually done in studies of isotopic turnover), a plateau is clearly indicated. In addition, our simulations showed that having a good coverage of the plateau was not required for estimating accurately half-life (Figure S2). The difference between the expected parameter estimates for the asymptote at 200 and 70 days were small and within isotopic measurement error. In addition, we obtained similar turnover estimates with or without the last point of sampling for blood cells (see below), further indicating that the model for estimating turnover estimates was not sensible to a visual criteria of equilibrium. Finally, compared to many of experimental studies with an average sample size of five, our study design with n = 40 allowed a gain in precision of close to 3 times (√8) for the estimation of the standard error of parameters. Overall, this showed that our results were robust for estimating discrimination and turnover.

For both isotopes in plasma and for δ^13^C in blood cells, the selected models included only individuals as a random effect for the slope parameter of the non-linear regression (Table S4). For δ^15^N in blood cells, the model included an effect of sex, age and their interaction. The mean half-lives of δ^13^C and δ^15^N differed by 3 and 5 days on average in blood cells and plasma, respectively (43 vs. 40 days in blood cells; 9 vs. 4 days in plasma; Table 2 & Figure 3). Compared to plasma half-life, which can vary up to 4 days among individuals, blood cells half-life shows considerable variability with up to approximately a month among individuals. In blood cells, we found that individual half-life could vary almost two times more than any level of grouping for both isotopes. Though the mean half-life in δ^13^C was rather similar across demographic categories, the mean half-life in δ^15^N was 5 days longer in yearlings compared to adults and 10 days longer in females compared to males (Table 2).

**Table 2:** Isotopic half-lives in arctic fox depending of the group (tissue, age, individuals) and the stable isotope (δ^13^C and δ^15^N).

	Half-life	Blood cells	Plasma
**δ^13^C**	All individuals	43 (28, 62)	9 (7, 11)
**δ^15^N**	Adult	38 (25, 55)	
	Yearling	43 (26, 71)	
	Female	46 (27, 76)	
	Male	36 (25, 52)	
	All individuals	40 (28, 67)	4 (3, 5)

Subscripts: N = 20 individuals arctic foxes. We give both average values and 95% confidence intervals within parentheses. For those isotopes and tissue types where model selection indicated no effect of sex and age, only estimates for all individuals are given.

## Discussion

Our multi-factorial experiment revealed that within a single species, isotopic discrimination was affected concomitantly and synergistically by several factors related to their diet and population structure. Based on relatively large sample size, we showed that such effects could be classified by decreasing order of importance as: isotopes, diet, tissue, age, and sex. Such results are difficult to compare with other experimental studies, so we do not know if this ranking has a wide applicability. We also demonstrated that turnover varied between isotopes and showed high variability among individuals. Furthermore, effects of sex and individual were for the first time observed as levels of population structure affecting discrimination and turnover in a consumer.

The importance of using multi-factorial experiments is evident when considering previous studies, which are providing mean values of discrimination for different diet types (e.g. [12], [13], [15], [16]). Since these studies used different individuals for each diet, the effect of diet composition was confounded with individual variation. Clearly, designs with replication both at levels within and among individuals, as done in the present study, are needed to partial out the effect of diet (i.e. food isotope ratio) from other factors that affect discrimination (e.g. age and sex).

### Added insights and new perspectives on discrimination

Across ca 130 species distributed in four different taxonomic classes, nine tissues, and more than 12 diet types, the range of discrimination values available from experimental studies is large, with Δ^13^C [−4 to 5.2‰] and Δ^15^N [−3.2 to 9.2‰] [4], [12], [13]. If we restrict such ranges to mammals only, it comes down to Δ^13^C [−0.5 to 4.5‰] and Δ^15^N [1.4 to 6.4‰]. In our study, we showed that, for a single species, in six tissues, and three diet types, the range can be large compared to the whole mammalian and avian classes, with [0.2‰ to 2.9‰] and [0.3‰ to 5.3‰] for Δ^13^C and Δ^15^N, respectively. Overall, this has several implications for diet reconstruction studies given the sensibility to discrimination values [9], [11]. One way to tackle such large variability for a single population would be to build upon the possible integration of prior information as well as the implementation of standard deviation for the discrimination for each food source in the new Bayesian frameworks available for models of mixtures (e.g. [10]). This will be a step forward to the current problem of combining discrimination for the whole, mixed diet without accounting for the variability inside the mixture (e.g. [17]).

Overall, the mean discrimination for both isotopes (Δ^13^C & Δ^15^N) was slightly lower than that derived from the literature review of Caut et al. [12]. However, predicted estimates from their models were mostly similar to the real estimates for the marine and terrestrial diet in the present study. This suggests that when designing an experiment for estimating discrimination factors, it is a better option to implement the types of experimental diet that are similar to those of wild animals rather than using a commercial mixture (See [4] and the related comments on this article). Using the two most extreme diet types, one can capture the whole range of diet isotope signatures, the consumer may encounter. Among tissues, our results follow the same pattern as found in previous animal experiments (e.g. [29], [30], [31]), with grouping of discrimination values for tissues with similar amino-acids composition, i.e. the external and keratinous tissues (nails, fur and feathers), and the internal tissues (e.g. muscle and liver).

So far, nine experiments have been published for carnivores' discrimination (reviewed in [5], [12], [13]). Surprisingly, it is the animal order that was the most thoroughly studied, with almost as many values available than the whole bird class itself. It is, however, rare to find in the literature values for several species in the same genus. Here we could compare our values for the arctic fox to the ones of red fox (*Vulpes vulpes*) [30]. Δ^13^C in both species were very similar, while Δ^15^N differed the most, being always lower in arctic foxes compared to red foxes (ca 1.2‰), except for the fur, which had almost identical values.

To our knowledge, there are only three experimental detections of an age effect on discrimination in animals [30], [32], [33]. While the previous studies showed an age effect only for nitrogen and three tissues (liver, fur, and muscle), here we detected an age effect in both isotopes and all different tissues sampled in both adults and yearlings (blood cells, fur, muscle, nail, and plasma). As compared to the higher discrimination for yearlings vs. adults in red foxes [30], we found the reverse for arctic foxes, a result similar to Sakano et al. (2005) for the sockeye salmon (*Oncorhynchus nerka*) and to Matthews & Mazumber (2008) for *Daphnia pulicaria*. In addition, for muscles, the age effect was more complex in arctic foxes, depending on the sex. The paucity of studies considering age effects hampers the exploration of underlying, mechanistic causes of the dichotomy between adults and yearlings. In fact, most studies measuring an age effect were performed in the field and bear confounding effects with e.g. diet changes (e.g. [34], [35], [36]). In addition, several experiments did not support any age effect, for instance, in two species of mussels [37], cattle [38], or peregrine falcon (*Falco peregrinus*) [23].

An age effect could be related to differential metabolic pathways or syntheses, with for example, a higher rate of protein accretion in yearlings compared to adults. In birds, elevated levels of urea or uric acids in blood in chicks compared to adults have been hypothesized as a potential explanation for depletion in the amount of ^15^N ([39] and references therein). Such effects, mostly due to protein turnover could then have the potential to drive several age effects [40]. However, the effect of plasma uric acid is likely to be minimal, as the amount of protein in plasma is three orders of magnitude higher than that of uric acid in birds (see discussion in [41]). Alternatively, a decreased efficiency in the use of nitrogen over the lifespan and various reproductive events has the potential to explain a higher δ^15^N in older vs. younger animals (e.g. [42]). For now, the ubiquitous trade-off between growth and reproductive development in vertebrates seems to be a promising and parsimonious working hypothesis that could also be explored to understand the sex effect detected in our study. Fruitful avenues could be directed to explore the impact of protein turnover and the costs of reproductive efforts [3].

### Added insights and new perspectives on turnover

Half-lives in both isotopes are usually considered as equal (e.g. [25]). In our study, this is not the case for plasma, where half-life of δ^15^N is two times shorter than for δ^13^C. To date, there are only two studies demonstrating different half-lives in the same tissue for two isotopes [2], [24]. These experiments concern whole blood in the garden warbler (*Sylvia borin*) and the canvasback (*Aythya valisineria*) and showed a half-life of δ^15^N two times longer than for δ^13^C. As suggested by Bearhop et al. [25], discrepancies between the two isotopes turnover could be tissue-dependent. We add here that population structure effects on turnover are also isotope and tissue-dependent, which is a novel aspect to take into account.

The observed difference between isotopes could be linked to different rates of proteins absorption compared to lipids and carbohydrates, both containing negligible levels of nitrogen compared to proteins [24]. Since our samples were corrected for lipids (normalization and extraction of lipids; see Methods S1 for further details), we could hypothesize that carbohydrate assimilation rate could be an underlying factor, though carbohydrates were ca 3 times less present in the diet than proteins (Table S1). However, one experiment on farmed arctic foxes showed rather similar digestibility values for proteins and carbohydrates (ca 88% vs. 85%) [43]. Although individuals were fed *ad libitum*, several amino acids could have been built from lipid reserves and not only from their bulk diet [44], [45]. Finally, isotopic routing could be an additional, hypothetic mechanism toward explaining such patterns; routing occurring when certain dietary macromolecules (e.g., protein) are preferentially used to build proteinaceous tissues, thus reducing the cost of *de novo* synthesis (e.g. [3] and references therein, [46]). Whatever the underlying metabolic pathway, the decoupling between carbon and nitrogen has consequences in the interpretation of data from field studies (see [47]), where sampling of plasma in individuals shifting between prey sources could bear different signals on a weekly basis [24].

### Insights for ecological monitoring

One key aspect when planning ecological studies using stable isotopes to study the trophic ecology of consumers is to target tissues with ecologically relevant variations in discrimination and turnover rates [3], [31], [48]. When the demographic composition of the population is unknown, using blood would be the best solution because age and sex effect are small. For turnover, plasma would be a first choice for getting a precise window of diet variation (ca one week), while blood cells would be an interesting alternative if individuals not sampled simultaneously are to be compared, thus buffering the possible variation due to short time changes in prey availability. Sampling whole blood provide a promising isotopic-clock with the ability to capture and compare two time-scales of dietary information per individual. Before using such values for other species to track change since a diet shift, we recommend precautions and simulations following Klaassen, Piersma, Korthals et al. [49].

In general, studies on turnover report only the mean half-life [5], though considerable variation among individuals may occur. In our study, individuals within the same age and sex group may exhibit up to two times shorter half-lives than others for blood cells, while this does not occur in plasma. Such variance in the half-life range, which can be tissue-specific [25], bears considerable consequences for field studies relying on the average estimates to separate different window of time. Indeed, the choice of a specific tissue to monitor diet could then also be directed by the variance in half-life. This will allow field studies to explore the whole spectrum of diet variation and allowing greater flexibility in the possible prey sources available for the target consumer. In that context, we recommend that further experimental studies provide in addition to the average half-life estimates, confidence intervals or standard deviations as well as the variance components (to estimate variation among individuals).

Overall, our study shows that population structure affects isotopic discrimination. For species like the arctic fox exhibiting large fluctuation in age structure both on a seasonal and multi-annual time-scale [50], not accounting for population structure may confound interpretations of temporal variation in isotopic signature at the population level. For instance, temporal changes in isotopic signatures owing to altered age structure could be interpreted (erroneously) as a change in population level consumption. Moreover, differences in isotope signatures between parents and young in a single breeding season would partly reflect different discrimination and not only eventual difference in age-specific diet. Such context is applicable for a wide-range of species experiencing fluctuating environment and various strategies of energy allocation.

Given the predicted, high sensitivity of mixing model to variation in discrimination factors [9], [11], the scale of variation identified in the present study bode a wide range of possible biases in diet reconstructions. For now, it is difficult to anticipate the outcomes of mixing models while accounting for this variation because the sensitivity of a mixing model isotopic shift depends both on the complexity of the ecosystem (number of sources) and on the relative positions of sources and consumers within the isotopic mixing space [51]. This urges for the use of the flexible, Bayesian approaches for mixing models, like the SIAR package in R [10]. For now, only fixed, standard deviation of discrimination can be implemented. Following our study, using the information about the consumer's population structure (e.g. sex ratio, age structure) could be another level of Bayesian priors to explore the effects of such variation in any given configuration of prey and consumer. Designing experiments linked to the ecological conditions present in the wild, as well as a better understanding of the underlying physiology may help understand how life-history traits and population structure can modulate diet incorporation.

## Materials and Methods

### Ethics statement

The use of live animals in our experiments at the Animal Production Experimental Centre at University of Life Sciences in Ås, Norway, was supervised by the Norwegian National Animal Research Authority. This public committee ensured that the use of our laboratory animals was necessary and that animal welfare was respected. The centre permit for the Norwegian National Animal Research Authority is No. 109. The experiment did not need an additional special approval because the animals were in a situation of normal production during the course of the experiment.

### Experimental design

The study was conducted during the period 21-June-2007 to 21-Jan-2008 at the Norwegian University of Life Sciences, Department of Animal and Aquacultural Sciences, Ås, Norway. The experimental animals comprised 40-farmed arctic foxes (*Vulpes lagopus*) with a 20/20 proportion of females/males and adult/yearling (Figure 1). Yearlings used in the experiment had almost achieved their adult size so that possible changes in isotopic composition could not be due to growth (Mean body mass ± SD: 8.6 kg ±1.1 kg; n = 40). Details on captivity conditions and diet are provided in Methods S1 and Table S3.

We followed a classic design for measuring diet discrimination and turnover of animals fed on a continuous diet ([1], [14]. See below for details on the feeding schedule). The main aspects of the study design were as follows: 1- A multifactorial design was employed to assess the relative importance of individual heterogeneity, age, sex and diet type (mix, terrestrial and marine). 2- Individuals were randomly assigned to a group shifted to another diet after a window of time, allowing the isotope signatures of individuals to be in equilibrium with their controlled diet. 3- Diet changed two times in a row for the same group of individuals, allowing three consecutive evaluations of discrimination on the same individuals.

### Sampling scheme and isotope analyses

Two kinds of tissues were sampled from the foxes, metabolic active and inactive. In decreasing order of turnover expected from the literature [5], the active tissue types were plasma/serum (hereafter, plasma), blood cells, liver, and muscle. Plasma and blood cells were obtained from whole blood through centrifugation immediately after sampling. Whole blood was sampled in the post-absorptive phase approximately 20 h after feeding to prevent any impact of digestive products on the isotope analyses of plasma. To represent the inactive tissues, we selected winter fur and nails, which for the former correspond to the diet during a limited period of growth or for the latter were slowly growing. Altogether, the tissue types provide different opportunities of study “contexts”. For instance, while liver and muscle can only be sampled on dead individuals, all the other tissues can be taken non-destructively. They can then be informative for monitoring purposes in the wild with limited impacts on the target species. All details on isotope measurements and laboratory procedures are provided in Methods S1. In total, we analyzed 1,256 fox samples over the course of the study.

Following our design, discrimination of blood tissues could be estimated for each of the three diet types (Figure 1). After the shift to the marine diet (called day “0”), individuals were sampled for blood on days 0, 4, 7, 11, 15, 19, 23, 28, 35, 55, and 70. Such repetitive sampling allowed the monitoring of blood turnover in the marine group. Simultaneously, the sampling of individuals kept on the terrestrial diet was a way to control whether the isotope ratios for blood changed over the course of the experiment as well as monitoring whether individuals achieved a stable isotopic ratio prior to their diet shift (Figure S1). Fur and nail were only sampled at the start of the experiment and discrimination was thus only estimated for the mix diet, on which they grew (Figure 1). For instance, nails were sampled at 2 cm from the tip (just before the blood vessel to prevent any pain); as they grow 1cm/month (unpublish. data), they clearly reflected the mix diet. We assumed that liver and muscle sampled at the end of the experiment represent equilibrium discrimination values based on the only available turnover estimates in the literature for mammals, ca 13 and 60 days for liver and muscle, respectively [1], [5]. Five individuals had to be removed from the experiment for various external reasons not related to our experiment, on days 40, 41, 52, 56, and 60. However, our total sample size remained equal to 40, as we used additional individuals fed on the same diet and kept as back up. The isotopic values of the individuals sacrificed earlier were similar to the individuals measured after 70 days for both isotopes for liver and muscle and did not show any temporal trend. We are therefore confident that we reached the equilibrium for both terrestrial (which remained identical for 140 days) and marine diet (which remained identical for 70 days) for these two tissues. In that context, discrimination for both tissues was estimated at day 70 for both marine and terrestrial diet types (Figure 1).

### Data analyses

All statistical analyses were implemented in R 2.10.1 [52], using in particular the ‘nlme’ [53] and ‘boot’ libraries. For residuals and random effects, normality was checked with the Lilliefors test. There was missing information for some individuals, which resulted in varying sample sizes in the different analyses. We focused our results on confidence intervals, as they are equivalent to a significance test but convey more information about the biological significance [54], [55].

Analyzing discrimination. Discrimination between a food resource and a consumer is described in terms of the difference in delta (δ) isotopes values (X) using the Δ notation, where
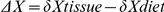



We examined the effect of diet, sex and age, and tissue on Δ of both isotopes using linear mixed effect models. Diet, sex, age, and tissue were modelled as fixed effects, while individuals were included as random effects. As different tissues were sampled in different phases of the experiment, not all tissues could be included into a single statistical analysis (see above). Due to limited sample size for liver, we could not assess the effect of all variables for this tissue and used Student t-statistic to test whether the Δ for both isotopes differed between the terrestrial and marine diet types.

Overall, we considered models with all effects and their relevant interactions as the most general model. Full and reduced models were fitted using maximum likelihood estimation; final parameters were estimated using the restricted maximum likelihood [56]. To select the most parsimonious model, we used the Akaike's Information Criterion (AIC with a cut-off of ΔAIC <2) and the relative weight of evidence in favour of a particular model (ωAIC) [57]. In presence of uncertainty in model selection, we chose the simplest model. Statistical significance of individual parameters was assessed using 95% confidence intervals. Model selection was only performed on the fixed effects, always including the estimation of the variance component due to individual heterogeneity.

Analyzing tissue turnover. Several experimental studies determined that isotopic turnover in blood is similar to an exponential decay function, possibly mimicking a first-order rate kinetic for the isotopic incorporation [3], [6]. This corresponds to the so-called one-compartment model [6], [28], [58]. To allow individual variation to be taken into account in such isotopic model, we used a non-linear mixed model implemented with the function nlme, fitting the following model to the raw values for each isotope and blood cells or plasma [59]:
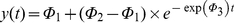
(1)where *Φ*_1_ is the asymptotic isotopic value, *Φ*_2_ the initial isotopic value and exp(*Φ*_3_) the slope of the regression. Half-life is then equal to 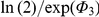.

Recently, multi-compartment modelling has been applied to the study of isotopic turnover [3], [6], [28], [58], [60]. In brief, this could lead to a better adjustment of kinetic curves and prevent a potential bias towards lower half-lives found in one vs. multicompartment model [28]. Following the equations in Martínez del Rio & Anderson-Sprecher [58] and Carleton et al. [28], we used a non-linear mixed model to allow the measure of individual heterogeneity. In addition, we also tried a simpler non-linear squares fit (i.e. ignoring the individual random effect) as well as a Bayesian approach to model both the one- and two-compartments for the isotopic turnover of plasma and blood cells inn arctic foxes. The latter was implemented using MCMC simulations implemented in WinBUGS and classical priors for the different parameters of the models and random effects [61]. Scripts are available on request.

Model selection was performed as described above with AIC. Competing models differ on the inclusion of random, sex or age effect on *Φ*_1_, *Φ*_2_, and *Φ*_3_. Random effect was always present at least in one of the *Φ*-parameters.

We furthermore used this statistical modelling framework to assess the robustness of parameter estimates to reducing the temporal span. We simulated isotopic values according to the asymptotic regression model given by Eq. 1, including random variation among individuals in parameter values, and using parameter values estimated from our data. We then sub-sampled times of observations to assess the bias and precision of parameter estimates (See Methods S1).

## Supporting Information

Methods S1 **Captivity conditions, sample preparation, isotopes measurement standards, turnover simulation and R scripts, and supplemental references.**
(DOC)

Figure S1 **Plasma and blood cells stable-carbon/nitrogen isotopes for the control group of arctic foxes.** One line trajectory corresponds to one individual (n = 20). See methods for details.
(TIF)

Figure S2 **Example of possible output from TurnoverSim, a R-script to model tissue turnover.** The script provides boxplots of the estimates of the three key parameters measuring turnover (asymptote, intercept and half-life) with different number of days of tissue sampling since diet shift. See Methods for further details.
(TIF)

Table S1 **Factors affecting discrimination between the diet and tissues of 40 arctic foxes.**
(DOC)

Table S2 **Factors affecting turnover in blood tissues of 20 farmed arctic foxes.**
(DOC)

Table S3 **Dietary composition and chemical content of the three experimental diet (in % otherwise mentioned) of arctic foxes, 2007-2008, Ås, Norway.**
(DOC)

Table S4 **Mean δ^13^C, δ^15^N, and CN ratio ± SD (‰) (in normal, italic, and bold fonts, respectively) of arctic foxes according to tissues, population structure and diet; 2007-2008, Ås, Norway.**
(DOC)
