# Supplementary material for: Intrapopulation Variability Shaping Isotope Discrimination and Turnover: Experimental Evidence in Arctic Foxes
Source: PLoS One. 2011 Jun 23;6(6):e21357. doi: 10.1371/journal.pone.0021357 (PMC3121787; doi:10.1371/journal.pone.0021357)
Supplement: Methods S1 — Captivity conditions, sample preparation, isotopes measurement standards, turnover simulation and R scripts, and supplemental references. (DOC) [file pone.0021357.s001.doc]

# Supporting methods S1

## Intrapopulation variability shaping stable isotope discrimination and turnover: experimental evidence in arctic foxes

## Nicolas Lecomte^1*^, Øystein Ahlstrøm^2^, Dorothée Ehrich^1^, Eva Fuglei^3^, Rolf A. Ims^1^ and Nigel G. Yoccoz^1^

**1** Department of Arctic and Marine Biology, University of Tromsø, N-9037 Tromsø, Norway

**2** Department of Animal & Aquacultural Sciences, Norwegian University of Life Science, N-1432 Ås, Norway

**3** Norwegian Polar Institute, Fram, N-9296 Tromsø, Norway

* Corresponding author. E-mail: nicolas.lecomte@uit.no

This file includes:

**Captivity conditions**

**Sample preparation**

**Isotopes measurement standards**

**Turnover simulation and R scripts**

**Supplemental references**

Captivity conditions

All individuals were obtained at the same time from a breeding line in Norway raised for fur production. The animals were housed individually in cages (0.8-1.2-1.1 m) made of plastic coated netting. Each cage was furnished with two boxes; a top box and nest box inside for shelter. The cages were also equipped with a resting platform, activity objects made of wood and a feeding board. Water was provided at all times via a semiautomatic system. The food was produced twice every week from the same pool of frozen mixture and kept in a refrigerator until fed. The animals were fed once every day and the food consumption was recorded on a group basis. Each food type was homogenized to prevent foxes from selecting one particular component of the diet mixture, which could have biased our estimates of discrimination.

The dichotomy between the terrestrial and marine diet types was set to maximize the isotopic difference as well as delineating two boundaries of the isotope spectrum used by wild foxes. A one-way ANOVA confirmed that the three diet types differed significantly in their isotopic composition (3 replicate samples of each diet type along the course of the study gave δ^13^C: *F*_2, 9_ = 101.84, P<0.001; δ^15^N: *F*_2, 9_ = 101.76, P<0.001). The range of the isotopic values was wide among diets (δ^13^C: -20.18, -27.10 and δ^15^N: 5.14, 11.35; Table S3). While the terrestrial diet was the most negative for δ^13^C, the marine diet stands out as the highest values for δ^15^N as expected. Although the isotopic signatures were different among the three diet types, the nutritional quality was the same. This is essential to prevent nutritional stress, which can cause changes in stable isotopes of consumers [see 1].

Foxes were regularly weighted to the nearest 0.02 kg to monitor the completion of *ad libitum* feeding. The average body mass during the experiment (See Material and Methods) suggested that animals were not nutritionally stressed by the experimental diet nor by their captivity conditions.

**Sample preparation**

Fur and nail samples were stored in paper bags until they were processed in the lab. Fur samples were washed using an ultrasonic bath and surface lipids were removed using a solution of 2:1 chloroform-methanol. After drying, the fur was cut to fine pieces with scissors. Nails were thoroughly washed, cleaned for surface lipids as described above and ground to powder with a nail file. Plasma, blood, muscle, liver and diet samples were frozen (-20°C) until they were freeze-dried, homogenized, and ground to a fine powder using a mixer mill (MM301; Retsch GmbH and Co., Haan) and carbide beads. Dried and powdered samples were weighed into tin capsules (precision +/- 0.01 mg). All these steps were conducted in the Department of Biology, University of Tromsø. Final isotopic analyses were then performed by the Stable Isotopes in Nature Laboratory (SINLAB), University of New Brunswick, Canada. Samples were combusted in a CarloErba NC2500 elemental analyzer connected via continuous-flow to a Finnigan Mat Delta Plus isotope-ratio mass-spectrometer (Thermo Finnigan, Bremen, Germany).

Since variations in lipid concentration of the tissue sample can significantly influence δ^13^C [e.g. 1,2], lipids were chemically removed from the bulk samples of diet, blood and liver through successive rinsing of powdered samples with 1 ml of 2:1 chloroform-methanol as a solvent, following the modified method of Bligh and Dyer [3]. The samples were then vigorously shaken several times and, after 15 min, centrifuged for 10 min. The supernatant was discarded and the procedure repeated at least once or until the supernatant was clear and colourless after centrifuging. As expected, lipid extracted samples of diet were less depleted in ^13^C than bulk samples (Tables 2 & S4 for corrected and uncorrected values, respectively). For economic reasons, we used a mathematical lipid correction (normalization) developed for muscle, a reliable alternative to chemical extraction, using the following equation [4]:

**Isotope measurement and standards**

Stable isotope ratios of carbon (δ^13^C) and nitrogen (δ^15^N) are expressed as permil (‰) of the deviation from isotopic ratios of international standards, i.e. Pee Dee Belemnite carbonate for carbon and atmospheric air for nitrogen.

X = [(R_sample_-R_standard_)/R_standard_] × 1000

where X (expressed in ‰) is δ^13^C or δ^15^N and R is the absolute isotopic ratio, respectively ^13^C/^12^C or ^15^N/^14^N.

Analytical error is reported by providing measures of precision and accuracy [5]. We evaluated the overall precision of measurement by randomly duplicating a subset of our samples: this includes both errors of precision inherent in the mass spectrometer and within-sample variations due to lack of homogeneity of powdered samples.

Average absolute difference between duplicates was 0.15‰ ± 0.05‰ SD (n = 40) for both δ^13^C and δ^15^N. Accuracy was estimated at SINLAB with measurements of a commercially available standard (Nicotinamide, Elemental Microanalysis Ltd.): δ^13^C = -34.2 ± 0.1‰ and δ^15^N = -1.8 ± 0.1‰ (n = 58). Precision was measured across runs using an internal smallmouth bass muscle standard: δ^13^C = -23.3 ± 0.1‰ SD and δ^15^N = 12.4 ± 0.2‰ SD (n = 23).

**Turnover simulation and R scripts**

This script, called TurnoverSim, was writen in R 2.11.1 [6]. This is simulation tool for stable isotope analyses to test whether experiments were able to achieve the requirements for measuring isotopic incorporation into tissues (i.e. equilibrium). See Fig. S2 for an example of a plot you can obtain with TurnoverSim. The script is detailed below and can also be found at ‘www.arctic-predators.uit.no’.

# simulations with TurnoverSim

nind=200

# Number of individuals

ndays=200

# Number of days

nt = nind*ndays

# Number of total data points

days=rep(1:ndays,times=nind)

str(days)

# days of sampling

id = factor(rep(1:nind,each = ndays))  # id of individuals

n.sim=200

mat.fix0=mat.fix1=mat.fix2=mat.fix3=mat.fix4=mat.fix5=array(0,dim=c(n.sim,3))

sd.res=0.2

mean.int= -24.2            # mean value of the yinit for each individual

mean.a= -4.2               # mean value of the slope

mean.asymp= -19.3          # mean value of the asymptotic value

days.sel0=c(1,10,20,30,40,50,70,90,110,150,200)

days.sel1=c(1,10,20,30,40,50,70,90,110,150)

days.sel2=c(1,10,20,30,40,50,70,90,110)

days.sel3=c(1,10,20,30,40,50,70,90)

days.sel4=c(1,10,20,30,40,50,70)

days.sel5=c(1,10,20,30,40,50)

for (i in 1:n.sim) {

intercept= rep(rnorm(nind, mean=mean.int, sd=0.2),each=ndays)

# simulated value of the yinit for each individual

a=rep(rnorm(nind, mean=mean.a, sd=0.01),each=ndays)

# simulated value of the slope

asymp=rep(rnorm(nind, mean=mean.asymp, sd=0.02),each=ndays)

# simulated value of the asymptotic value

d13c = asymp+(intercept-asymp)*exp(-exp(a)*(1:ndays)) + rnorm(n=nt, 0 , sd=sd.res)

aas=as.data.frame(cbind(days,id,d13c))

mat.fix0[i,]=fixed.effects(nlme(d13c ~ SSasymp(days,Asym,R0,lrc), fixed=Asym+R0+lrc~1, random=R0~1|id, start=c(Asym=-15,R0=-25,lrc=-4),data=aas[which(aas$days %in% days.sel0),]))

mat.fix1[i,]=fixed.effects(nlme(d13c ~ SSasymp(days,Asym,R0,lrc), fixed=Asym+R0+lrc~1, random=R0~1|id, start=c(Asym=-15,R0=-25,lrc=-4),data=aas[which(aas$days %in% days.sel1),]))

mat.fix2[i,]=fixed.effects(nlme(d13c ~ SSasymp(days,Asym,R0,lrc), fixed=Asym+R0+lrc~1, random=R0~1|id, start=c(Asym=-15,R0=-25,lrc=-4),data=aas[which(aas$days %in% days.sel2),]))

mat.fix3[i,]=fixed.effects(nlme(d13c ~ SSasymp(days,Asym,R0,lrc), fixed=Asym+R0+lrc~1, random=R0~1|id, start=c(Asym=-15,R0=-25,lrc=-4),data=aas[which(aas$days %in% days.sel3),]))

mat.fix4[i,]=fixed.effects(nlme(d13c ~ SSasymp(days,Asym,R0,lrc), fixed=Asym+R0+lrc~1, random=R0~1|id, start=c(Asym=-15,R0=-25,lrc=-4),data=aas[which(aas$days %in% days.sel4),]))

mat.fix5[i,]=fixed.effects(nlme(d13c ~ SSasymp(days,Asym,R0,lrc), fixed=Asym+R0+lrc~1, random=R0~1|id, start=c(Asym=-15,R0=-25,lrc=-4),data=aas[which(aas$days %in% days.sel5),]))

}

apply(mat.fix1,MAR=2,mean)

apply(mat.fix1,MAR=2,sd)

apply(mat.fix2,MAR=2,mean)

apply(mat.fix2,MAR=2,sd)

apply(mat.fix3,MAR=2,mean)

apply(mat.fix3,MAR=2,sd)

par(mfrow=c(3,1))

boxplot(mat.fix0[,1],mat.fix1[,1],mat.fix2[,1],mat.fix3[,1],mat.fix4[,1],mat.fix5[,1],names=c("200","150","110","90","70","50"),las=1)

abline(h=mean.asymp); title("Asymptote")

boxplot(mat.fix0[,2],mat.fix1[,2],mat.fix2[,2],mat.fix3[,2],mat.fix4[,2],mat.fix5[,2],names=c("200","150","110","90","70","50"),las=1)

abline(h=mean.int); title("Intercept")

boxplot(log(2)/exp(mat.fix0[,3]),log(2)/exp(mat.fix1[,3]),log(2)/exp(mat.fix2[,3]),log(2)/exp(mat.fix3[,3]),log(2)/exp(mat.fix4[,3]),log(2)/exp(mat.fix5[,3]),

names=c("200","150","110","90","70","50"),las=1)

abline(h=log(2)/exp(mean.a)); title("Half-life")

#end of the simulations. Further updates of the scripts would be available at www.arctic-predators.uit.no

## Supplemental references
