# Supplementary figures and images for: Intrapopulation Variability Shaping Isotope Discrimination and Turnover: Experimental Evidence in Arctic Foxes
Source: PLoS One. 2011 Jun 23;6(6):e21357. doi: 10.1371/journal.pone.0021357 (PMC3121787; doi:10.1371/journal.pone.0021357)

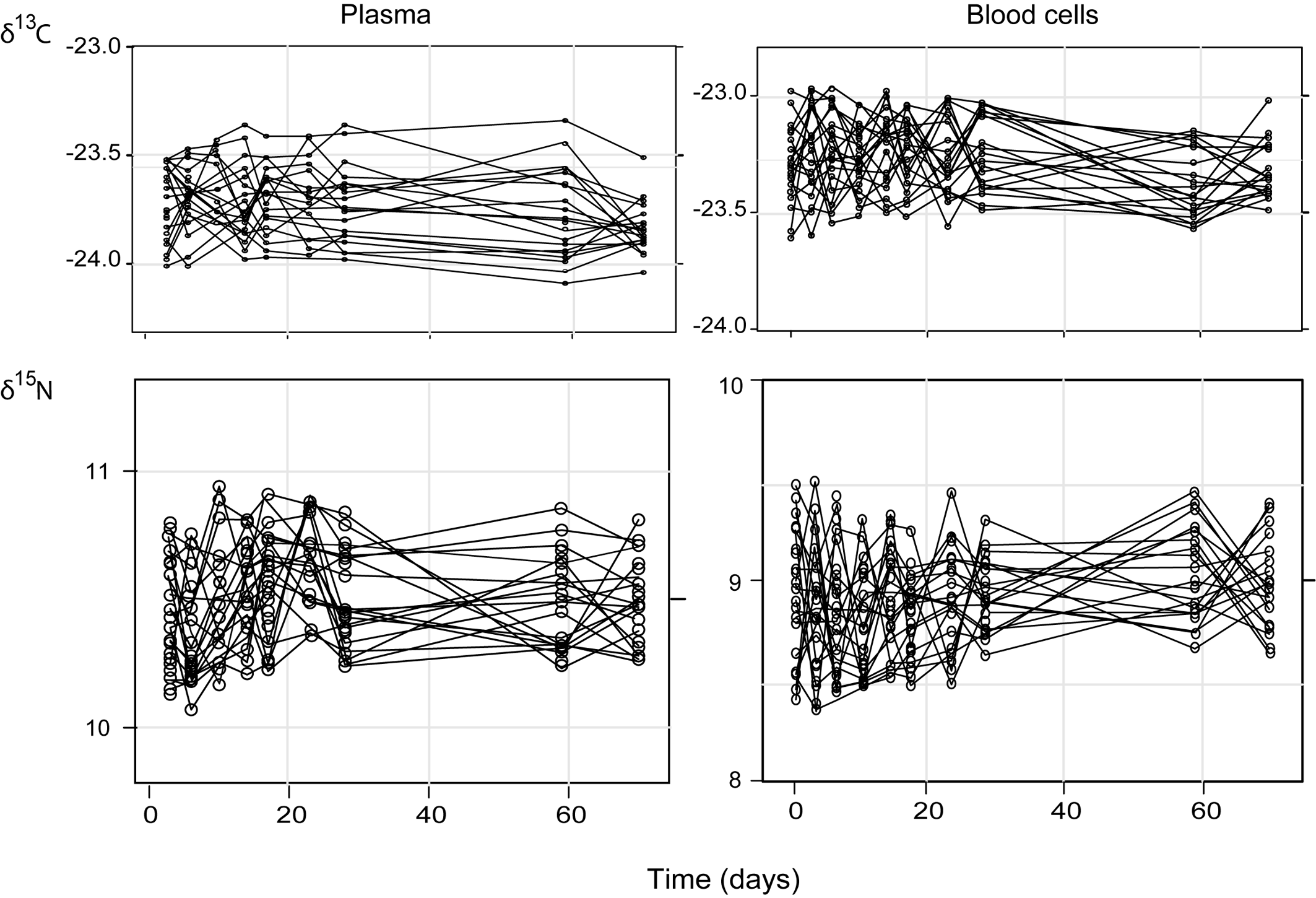

Supplement: Figure S1 — Plasma and blood cells stable-carbon/nitrogen isotopes for the control group of arctic foxes. One line trajectory corresponds to one individual (n = 20). See methods for details. (TIF) [file pone.0021357.s002.tif]

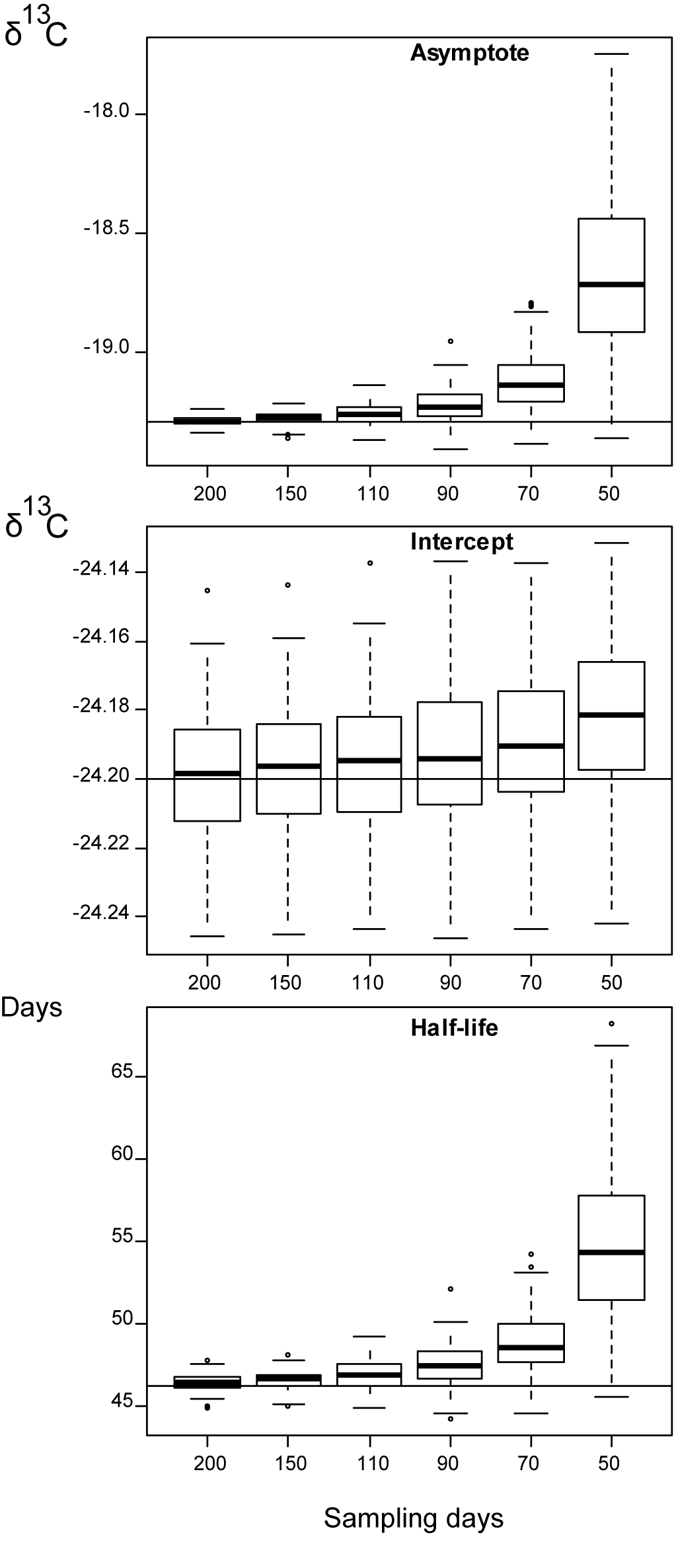

Supplement: Figure S2 — Example of possible output from TurnoverSim, a R-script to model tissue turnover. The script provides boxplots of the estimates of the three key parameters measuring turnover (asymptote, intercept and half-life) with different number of days of tissue sampling since diet shift. See Methods for further details. (TIF) [file pone.0021357.s003.tif]
