# Supplementary material for: Intrapopulation Variability Shaping Isotope Discrimination and Turnover: Experimental Evidence in Arctic Foxes
Source: PLoS One. 2011 Jun 23;6(6):e21357. doi: 10.1371/journal.pone.0021357 (PMC3121787; doi:10.1371/journal.pone.0021357)
Supplement: Table S1 — Factors affecting discrimination between the diet and tissues of 40 arctic foxes. (DOC) [file pone.0021357.s004.doc]

# Supporting Information

## Intrapopulation variability shaping stable isotope discrimination and turnover: experimental evidence in arctic foxes

## Nicolas Lecomte^1*^, Øystein Ahlstrøm^2^, Dorothée Ehrich^1^, Eva Fuglei^3^, Rolf A. Ims^1^ and Nigel G. Yoccoz^1^

**1** Department of Arctic and Marine Biology, University of Tromsø, N-9037 Tromsø, Norway

**2** Department of Animal & Aquacultural Sciences, Norwegian University of Life Science, N-1432 Ås, Norway

**3** Norwegian Polar Institute, Fram, N-9296 Tromsø, Norway

* Corresponding author. E-mail: nicolas.lecomte@uit.no

**Table S1.** Factors affecting discrimination between the diet and tissues of 40 arctic foxes.

|  |  | Tissues | | | | | | | | |  |
| --- | --- | --- | --- | --- | --- | --- | --- | --- | --- | --- | --- |
|  |  | Active | | | | |  | | Inactive | |  |
| Δ^13^C | Factors | Blood cells & Plasma^a^ |  | Liver |  | Muscle | |  | | Fur & Nail^a^ | |
|  | Age (Yearling) | -0.1 (-0.3, 0.1) |  | X |  | **-0.3 (-0.6, -0.1)** | |  | | **-0.6 (-0.8, -0.4)** | |
|  | Sex (Males) | -0.1 (-0.2, 0.1) |  | X |  | -0.2 (-0.4, 1.1) | |  | | -0.4 (-0.6, 0.2) | |
|  | Age (Yearling) * Sex (Males) | 0.1 (-0.1, 0.2) |  | X |  | **0.3 (0.1, 0.7)** | |  | | **0.3 (0.0, 0.5)** | |
|  | Tissue ^a^ | **0.3 (0.2, 0.3)** |  | X |  | X | |  | | **0.3 (0.1, 0.5)** | |
|  | Tissue (Nail) * Age (Yearling) | X |  | X |  | X | |  | | **-0.5(-0.8, -0.3)** | |
|  | Diet (Mix) | -0.0 (-0.2, 0.1) |  | X |  | X | |  | | X | |
|  | Diet (Terrestrial) | 0.0 (-0.14, 0.18) |  | -0.1(0.4, 0.1) |  | **1.2 (1.1, 1.4)** | |  | | X | |
|  | Diet (Mix) * Age (Yearling) | **-0.3 (-0.6, -0.2)** |  | X |  | X | |  | | X | |
|  | Diet (Terrestrial) * Age (Yearling) | -0.3 (-0.1, 0.1) |  | X |  | X | |  | | X | |
|  | Among-individual standard deviation ^b^ | Not estimable ^c^ |  | X |  | X | |  | | X | |
|  | Within-individual standard deviation (residuals) ^b^ | **0.2 (0.2, 0.3)** |  | X |  | X | |  | | X | |

Subscripts: Estimates are presented with their 95 % confidence interval (in bold when the interval does not include 0). The reference levels are age adult, diet marine, sex females.

**Table S1.** continued

| Δ^15^N | Age (Yearling) | 0.2 (-0.1, 0.5) |  | X |  | 0.0 (-0.2, 0.2) |  | **-0.9 (-1.3, -0.9)** |
| --- | --- | --- | --- | --- | --- | --- | --- | --- |
|  | Sex (Males) | -0.0 (-0.2, 0.1) |  | X |  | 0.2 (-0.0, 0.4) |  | 0.4 (-0.0, 0.9) |
|  | Age (Yearling) * Sex (Males) | -0.0 (-0.3, 0.2) |  | X |  | **-0.4 (-0.6, -0.1)** |  | -0.4 (-0.9, 0.1) |
|  | Tissue ^a^ | **1.5 (1.3, 1.6)** |  | X |  | X |  | 1.0 (0.68, 1.4) |
|  | Tissue (Nail) * Age (Yearling) | X |  | X |  | X |  | **-1.4 (-1.9, -0.9)** |
|  | Diet (Mix) | **1.8 (1.5, 2.0)** |  | X |  | X |  | X |
|  | Diet (Terrestrial) | **0.8 (0.6, 1.1)** |  | **0.72 (0.23, 1.20)** |  | **1.5 (1.4, 1.7)** |  | X |
|  | Diet (Mix) * Age (Yearling) | **-0.6 (-0.9, -0.6)** |  | X |  | X |  | X |
|  | Diet (Terrestrial) * Age (Yearling) | -0.3 (-0.7, 0.3) |  | X |  | X |  | X |
|  | Among-individual standard deviation ^b^ | Not estimable ^c^ |  | X |  | X |  | X |
|  | Within-individual standard deviation (residuals) ^b^ | **0.4 (0.3, 0.4)** |  | X |  | X |  | X |

Subscripts: a. Reference levels are blood cells and fur. b. Random terms involving only the constant term (i.e. random intercepts fitted per individual). Within-individual variation sums up all the possible sampling and measurement errors. c. With only 3 values for the same individuals, our design did not allow the direct measurement of the individual effect. X. No such effect
