# Supplementary material for: Intrapopulation Variability Shaping Isotope Discrimination and Turnover: Experimental Evidence in Arctic Foxes
Source: PLoS One. 2011 Jun 23;6(6):e21357. doi: 10.1371/journal.pone.0021357 (PMC3121787; doi:10.1371/journal.pone.0021357)
Supplement: Table S2 — Factors affecting turnover in blood tissues of 20 farmed arctic foxes. (DOC) [file pone.0021357.s005.doc]

# Supporting Information

## Intrapopulation variability shaping stable isotope discrimination and turnover: experimental evidence in arctic foxes

## Nicolas Lecomte^1*^, Øystein Ahlstrøm^2^, Dorothée Ehrich^1^, Eva Fuglei^3^, Rolf A. Ims^1^ and Nigel G. Yoccoz^1^

**1** Department of Arctic and Marine Biology, University of Tromsø, N-9037 Tromsø, Norway

**2** Department of Animal & Aquacultural Sciences, Norwegian University of Life Science, N-1432 Ås, Norway

**3** Norwegian Polar Institute, Fram, N-9296 Tromsø, Norway

* Corresponding author. E-mail: nicolas.lecomte@uit.no

**Table S2.** Factors affecting turnover in blood tissues of 20 farmed arctic foxes.

|  | Tissues | | | | |
| --- | --- | --- | --- | --- | --- |
| Factors | Blood cells | |  | Plasma | |
|  | δ^13^C | δ^15^N |  | δ^13^C | δ^15^N |
| Asymptotic value | **-19.5**  **(-20.1, -18.9)** | **12.3**  **(11.8, 12.7)** |  | **-20.2**  **(-20.3, -20.2)** | **13.7**  **(13.6, 13.8)** |
| Starting value | **-23.2**  **(-23.3, -23.2)** | **8.7**  **(8.7, 8.8)** |  | **-23.1**  **(-23.1, -23.0)** | **8.8**  **(8.6, 8.8)** |
| Individual slopes^a^ | **4.0**  **(3.8, 4.2)** | **3.9**  **(3.7, 4.1)** |  | **2.5**  **(2.5, 2.5)** | **1.8**  **(1.8, 1.8)** |
| Within-individual standard deviation (residuals)^a^ | **0.3**  **(0.2, 0.3)** | **0.3**  **(0.2, 0.4)** |  | **0.2**  **(0.1, 0.2)** | **0.3**  **(0.3, 0.4)** |

Subscripts: Estimates are presented with their 95% confidence interval (all estimates are in bold as their interval does not include 0). a. Random terms involving only the constant term (i.e. random intercepts fitted per individual). Within-individual variation sums up all the possible sampling and measurement errors.
