# Supplementary material for: Intrapopulation Variability Shaping Isotope Discrimination and Turnover: Experimental Evidence in Arctic Foxes
Source: PLoS One. 2011 Jun 23;6(6):e21357. doi: 10.1371/journal.pone.0021357 (PMC3121787; doi:10.1371/journal.pone.0021357)
Supplement: Table S3 — Dietary composition and chemical content of the three experimental diet (in % otherwise mentioned) of arctic foxes, 2007-2008, Ås, Norway. (DOC) [file pone.0021357.s006.doc]

# Supporting Information

## Intrapopulation variability shaping stable isotope discrimination and turnover: experimental evidence in arctic foxes

## Nicolas Lecomte^1*^, Øystein Ahlstrøm^2^, Dorothée Ehrich^1^, Eva Fuglei^3^, Rolf A. Ims^1^ and Nigel G. Yoccoz^1^

**1** Department of Arctic and Marine Biology, University of Tromsø, N-9037 Tromsø, Norway

**2** Department of Animal & Aquacultural Sciences, Norwegian University of Life Science, N-1432 Ås, Norway

**3** Norwegian Polar Institute, Fram, N-9296 Tromsø, Norway

* Corresponding author. E-mail: [nicolas.lecomte@uit.no](mailto:nicolas.lecomte@uit.no)

**Table S3.** Dietary composition and chemical content of the three experimental diet (in % otherwise mentioned) of arctic foxes, 2007-2008, Ås, Norway.

| **Diet composition** | **Diet** | | |
| --- | --- | --- | --- |
| **Items** | **Mix** | **Marine** | **Terrestrial** |
| Capelin, whole | X | 30 | X |
| DL-methionine | 0.1 | X | X |
| Fish scraps, cod and salmon | 7.5 | X | X |
| Fishmeal, Norseamink | 3.5 | 5 | X |
| Gras meal | 0.5 | X | X |
| Household waste | 5 | X | X |
| Meat-and-bone meal | 10 | X | 9.2 |
| Poultry by-products | 50.9 | X | 23 |
| Precooked carbohydrates, barley-wheat | 10 | 6 | 3.7 |
| Salmon oil | X | 5 | X |
| Seal oil | 0.5 | X | X |
| Slaughterhouse by-products, cattle | 9.1 | X | 50.6 |
| Vitamin-mineral mixture | 0.1 | 0.1 | 0.1 |
| Water | 2.9 | 17.9 | 13.4 |
| Whale meat, minke | X | 36 | X |
| **Chemical content** |  |  |  |
| Dry matter g/kg | 43.2 | 34.7 | 37.5 |
| Ash g/kg | 5.3 | 1.8 | 4.3 |
| Protein g/kg | 17 | 17.6 | 13 |
| Fat g/kg | 11.4 | 10.7 | 17.2 |
| Carbohydrates (by difference) g/kg | 9.5 | 4.6 | 3 |
| Metabolizable energy content kJ/kg | 7600 | 7070 | 8500 |
| MJ/kg per kg dry matter | 17.6 | 20.4 | 22.7 |
| Metabolizable energy distribution, | 34-53-14 | 38-54-7 | 24-72-4 |
| % from protein, fat and carbohydrates |
| **Isotopes (mean ± SD)** |  |  |  |
| **δ^13^C ‰** | -24.69 ± 0.24 | -22.60 ±0.54 | -26.75 ± 0.34 |
| **δ^15^N ‰** | 6.22 ± 0.37 | 10.98 ± 0.86 | 7.33 ± 0.66 |
| **δ^13^Cext ‰** | -22.64 ± 0.39 | -20.70 ± 0.49 | -23.76 ± 0.10 |
| **δ^15^Next ‰** | 6.19 ± 0.93 | 11.07 ± 0.22 | 7.56 ± 0.42 |
| **CN ratio** | 4.09 ± 0.24 | 3.71 ± 0.22 | 3.73 ± 0.15 |

Subscripts: δ ^13^Cext and δ^15^Next correspond to δ ^13^C and δ^15^N obtained after lipid extraction (see methods for details). Isotope measurements were made on 6 sub-samples of each diet. Chemical content of diet types were analyzed by standard methods at the Norwegian Fur Breeders Association Laboratory, Oslo. The two types of experimental diet were planned to have similar levels of protein, fat and carbohydrates. Chemical analyses revealed that the diets were somewhat different regarding the main nutrients. The terrestrial contained (g/kg) 17.6 % protein, 10.7 % fat and 4.6 % carbohydrates, while the marine contained 13.0, 17.2 and 3.0 %, respectively (Table 1S). However, both diets met the nutrient requirement for arctic foxes at their life stage during the experiment.
