# Supplementary material for: Intrapopulation Variability Shaping Isotope Discrimination and Turnover: Experimental Evidence in Arctic Foxes
Source: PLoS One. 2011 Jun 23;6(6):e21357. doi: 10.1371/journal.pone.0021357 (PMC3121787; doi:10.1371/journal.pone.0021357)
Supplement: Table S4 — Mean δ13C, δ15N, and CN ratio ± SD (‰) (in normal, italic, and bold fonts, respectively) of arctic foxes according to tissues, population structure and diet; 2007-2008, Ås, Norway. (DOC) [file pone.0021357.s007.doc]

# Supporting Information

## Intrapopulation variability shaping stable isotope discrimination and turnover: experimental evidence in arctic foxes

## Nicolas Lecomte^1*^, Øystein Ahlstrøm^2^, Dorothée Ehrich^1^, Eva Fuglei^3^, Rolf A. Ims^1^ and Nigel G. Yoccoz^1^

**1** Department of Arctic and Marine Biology, University of Tromsø, N-9037 Tromsø, Norway

**2** Department of Animal & Aquacultural Sciences, Norwegian University of Life Science, N-1432 Ås, Norway

**3** Norwegian Polar Institute, Fram, N-9296 Tromsø, Norway

* Corresponding author. E-mail: nicolas.lecomte@uit.no

**Table S4.** Mean δ^13^C, δ^15^N, and CN ratio ± SD (‰) (in normal, italic, and bold fonts, respectively) of arctic foxes according to tissues, population structure and diet; 2007-2008, Ås, Norway.

|  |  | Tissues | | | | | | |
| --- | --- | --- | --- | --- | --- | --- | --- | --- |
|  |  | Soft | | | |  | Hard | |
| Sex | Age | Blood cells | Plasma | Liver | Muscle |  | Fur | Nail |
| F | Y | -22.78 ± 0.63  *9.28 ± 0.70* | -23.34 ± 1.08  *9.93 ± 1.33* | - | -23.65 ± 1.33  *10.95 ± 1.20* |  | -20.66 ± 0.16  *9.04 ± 0.25* | -20.92 ± 0.32  *8.64 ± 0.32* |
|  | A | -22.51 ± 0.63  *9.64 ± 0.54* | -22.83 ± 0.85  *9.94 ± 1.23* | - | -24.16 ± 1.33  *10.81 ± 0..35* |  | -19.99 ± 0.22  *9.98 ± 0.38* | -19.70 ± 0.32  *10.89 ± 0.85* |
| M | Y | -22.74 ± 0.63  *9.09 ± 0.85* | -22.83± 0.70  *10.41 ± 1.01* | - | -23.73 ± 1.26  *10.91 ± 1.04* |  | -20.75 ± 0.13  *8.96 ± 0.32* | -21.06 ± 0.22  *8.66 ± 0.38* |
|  | A | -22.44 ± 0.63  *9.33 ± 0.85* | -22.62 ± 0.79  *10.48 ± 0.95* | - | -23.50 ± 1.01  *11.04 ± 0.54* |  | -20.48 ± 0.85  *10.22 ± 0.54* | -19.97 ± 0.32  *11.48 ± 0.82* |
| Overall |  | -22.62 ± 0.41 (40)  *9.34 ± 0.45*  **3.45 ± 0.10** | -22.90 ± 0.64 (40)  *10.19 ± 0.82*  **4.03 ± 0.69** | -22.78 ± 2.36 (16)  *11.36 ± 1.88*  **4.0 ± 0.34** | -23.74 ± 1.26 (40)  *10.93 ± 0.82*  **5.51 ± 0.81** |  | -20.46 ± 0.44 (39)  *9.53 ± 0.69*  **2.96 ± 0.06** | -20.45 ± 0.64 (34)  *9.79 ± 0.73*  **2.81 ± 0.12** |
| Overall  Mix |  | -22.36 ± 0.21 (17)  *8.72 ± 0.37* | -22.73 ± 0.29 (17)  *8.70 ± 1.53* | - | - |  | -20.46 ± 0.44 (39)  *9.53 ± 0.69* | -20.45 ± 0.64 (34)  *9.79 ± 0.73* |
| Overall  Mar |  | -20.55 ± 0.21 (17)  *11.45 ± 0.16* | -20.21 ± 0.16 (17)  *13.87 ± 0.29* | -20.63 ± 0.57 (8)*  *13.04 ± 0.62* | -22.51 ± 0.49 (20)*  *12.03 ± 0.27* |  | - | - |
| Overall  Terr |  | -23.45 ± 0.16 (17)  *9.06 ± 0.12* | -23.88 ± 0.49 (17)  *9.28 ± 0.78* | -24.21 ± 0.28 (8)*  *10.25 ± 0.28* | -24.78 ± 0.49 (20)*  *10.00 ± 0.22* |  | - | - |

Subscripts: Sample sizes are within brackets. A: adult; F: female; M: male; Y: yearling; * assuming that the delay since diet shift was long enough to exceed the turnover of both liver and muscle, following other estimates available from other mammals [7]
